# Effect of Primary Systemic Therapy on *PD-1, PD-L1*, and *PD-L2 mRNA* Expression in Advanced Breast Cancer

**DOI:** 10.31557/APJCP.2021.22.7.2069

**Published:** 2021-07

**Authors:** Ramadhan Karsono, Muhammad Al Azhar, Yulia Pratiwi, Fahreza Saputra, Siti Nadliroh, Teguh Aryandono

**Affiliations:** 1 *Department of Surgical Oncology, Dharmais National Cancer Center Hospital, Jakarta, Indonesia. *; 2 *Department of Research and Development, Dharmais National Cancer Center Hospital, Jakarta, Indonesia. *; 3 *Department of Functional Medical Staff of Surgical Oncology, Dharmais National Cancer Center Hospital, Indonesia. *; 4 *Department of Surgery, Faculty of Medicine Public Health and Nursing, Universitas Gadjah Mada, Indonesia. *

**Keywords:** PD-1, PD-L1, PD-L2, breast cancer, systemic therapy, immunotherapy

## Abstract

**Objective:**

The association between *PD-1, PD-L1*, and *PD-L2* expression and prognosis has been extensively studied in various cancers but remained controversial in breast cancer. Besides, little is known about the prognostic value of PD-1, PD-L1, and PD-L2 upregulation or downregulation following systemic therapy (chemotherapy and hormonal therapy) in breast cancer. Therefore, we aim to investigate the change of *PD-1, PD-L1*, and *PD-L2* expression in mRNA level after primary systemic therapy in breast cancer patients and its clinical implications.

**Methods:**

Expression of *PD-1, PD-L1*, and *PD-L2 mRNA *were measured before-after chemotherapy and hormonal therapy with real-time PCR in 80 advanced breast cancer patients. The correlation between alteration of *PD-1, PD-L1*, and *PD-L2* expression and clinicopathological characteristics as well as overall survival was also statistically analyzed.

**Results:**

Chemotherapy and hormonal therapy altered *PD-1, PD-L1*, and *PD-L2* expression in breast cancer with most patients have an increase expression. As much as 57.1%, 62.9% and 60% patients have an increase* PD-1, PD-L1,* and *PD-L2* expression after chemotherapy, while 60%, 60%, and 64% patients have an increase *PD-1, PD-L1*, and *PD-L2* expression after hormonal therapy. Alteration of* PD-1, PD-L1*, and *PD-L2* expression was not correlated with all clinicopathological characteristics. Increase in *PD-1, PD-L1*, and *PD-L2* expression was significantly associated with better OS (p=0.031, p=0.019, and p=0.019 for PD-1, PD-L1, and PD-L2, respectively), which remained significant in multivariate analysis including age, stage, primary systemic therapy, histology grade, subtype and primary tumor histology (HR PD-1 0.5 (95% CI 0.28-0.88) p=0.031; HR PD-L1 0.43 (95% CI 0.24-0.8) p=0.019; HR PD-L2 (95% CI 0.24-0.87) p=0.019).

**Conclusion:**

Expression of* PD-1, PD-L1*, and *PD-L2* in breast cancer patients is mostly enhanced after chemotherapy and hormonal therapy, and the enhancement is associated with good OS. This result revealed the potential of measuring *PD-1, PD-L1*, and *PD-L2 mRNA* expression in predicting clinical outcome.

## Introduction

Breast cancer has a high incidence and mortality rate and is estimated to be the most common malignancy in females worldwide. Every year incidences of breast cancer increase by more than 5%. In developing countries including Indonesia, most breast cancers were detected in advanced stages (3 and 4). Moreover, death is higher in low development countries (Ghoncheh et al., 2016; Youlden et al., 2014; Yuan et al., 2019). Types of therapies commonly used to treat breast cancer are radiotherapy, surgery, chemotherapy, hormone therapy, and targeted therapy. However, these therapies are not effective enough to treat breast cancer (Zhang et al., 2017). 

Currently there is a growing interest of immunotherapy in cancer treatment. The use of immune checkpoint inhibitors especially anti PD-1/ PD-L1 becomes the most popular immunotherapeutic strategy in recent treatment (Esteva et al., 2019). Anti PD-1/ PD-L1 also has shown good clinical effect in treatment of breast cancer (Planes-Laine et al., 2019). Programmed death 1 (PD-1) is a protein receptor expressed by T cell, B cell, and other immune cells. It has 2 ligands including programmed death ligand 1 (PD-L1) and programmed death ligand 2 (PD-L2) (Pardoll, 2012; Topalian et al., 2016). Expression of *PD-L1* is highly expressed in cancer patients (Azhar et al., 2020; Wang et al., 2016) and associated with poor prognosis in several cancer types (Zhang et al., 2017). However, *PD-L1* expression in breast cancer is still controversial with some studies reporting conflicting results (Schalper et al., 2014; Zhang et al., 2017).

Positive *PD-L1* expressions correlated with good response to immune checkpoint inhibitor therapy (anti-PD-1/PD-L1). Currently, measuring *PD-L1 *expression using immunohistochemistry (IHC) has been used to determine type of patients who respond immune checkpoint inhibitor therapy. However, the use of PD-L1 IHC has several limitations such as different cut offs, different scoring systems, variable detection antibodies, and processing variability (Bertucci et al., 2015; Patel and Kurzrock, 2015). This could affect the result which leads to conflicting data in several studies (Schalper et al., 2014). Therefore, the use of alternative method such as real-time PCR to assess *PD-L1* expression may help to overcome such limitations. It has been shown that there is positive correlation between PD-L1 protein and *mRNA *expression, which indicated potential of measuring *PD-L1 mRNA* expression to assess response to anti-PD-1/PD-L1 therapy (Kim et al., 2018). 

Chemotherapy and hormonal therapy are type of systemic therapy that majorly used to treat breast cancer patients in advanced stages. The idea of combining immune checkpoint inhibitor therapy with chemotherapy or hormonal therapy has been proposed to enhance the response rate and duration and improve survival (Esteva et al., 2019; Hühn et al., 2019; Luo and Fu, 2016; Page et al., 2019). Several studies reported that some chemotherapy agents could change *PD-L1* expression (Luo and Fu, 2016). On the other hand, the study of hormone therapy effect to* PD-1, PD-L1*, or *PD-L2* expression is very rare. To investigate the potential benefit of combination therapy between hormonal therapy/chemotherapy and immunotherapy, it is crucial to understand the impact of chemotherapy or hormonal therapy to *PD-L1* expression as well as* PD-1* and *PD-L2* expression. Besides, little is known about the prognostic value of PD-1, PD-L1, and PD-L2 upregulation or downregulation following systemic therapy in breast cancer. Therefore, in this study, we aimed to assess *PD-1, PD-L1, PD-L2 mRNA* expression before-after primary systemic therapy (chemotherapy and hormonal therapy) using real-time PCR and investigate the association with clinicopathological features and overall survival of advanced breast cancer patients. 

## Materials and Methods

*Patients’ samples collection and therapy given*

This study was a retrospective study conducted from 2011 to 2017 (n=80) at Dharmais National Cancer Center Hospital, Indonesia. Patients’ tumor tissues of advanced breast cancer (stages 3B and 4) patients were taken before and after primary systemic therapy (chemotherapy and hormonal therapy). Tissue samples were divided into two pieces, one piece for histological examination and another piece was directly put in cryotubes containing 1 mL of RNAlater then stored at -80^o^C to keep RNA integrity and quality. Criteria for stage 3B and 4 was determined based on American Joint Committee on Cancer 7^th^ edition guideline (American Cancer Society, 2010).

From 80 samples, 35 patients were received primary chemotherapy and 45 patients received primary hormonal therapy. All patients were given systemic therapy before the patient undergone surgery. Hormonal therapy group received Aromatase Inhibitor, Tamoxifen or GNRH-analogue during 6 months of treatment. The chemotherapy group received FAC (5-Fluorouracil, Adriamycin, and Cyclophosphamide) which were given for 6 cycles. 

Patients who have a mastectomy before primary systemic therapy, pregnant, and refuse to participate were excluded. The patient followed up was done continuously to obtain data of death, censored patients, and patients with new symptoms. All patients agreed to be involved in this study after signing informed consent. This study was approved by Ethical Committee at Dharmais Hospital-National Cancer Center, Indonesia (Number of Ethical Clearance: 9/KEPK/II/2019 and 10/KEPK/II/2019).

*Extraction of total RNA and cDNA synthesis*

Isolation of total RNA from tissue samples was done using RNA Tissue Mini Kit (Qiagen) according to manual instruction book provided by the kit. The total RNA was then measured for its concentration and purity using Nanodrop spectrophotometer. Maximum 2,000 ng of RNA was reverse transcribed to cDNA using High Capacity cDNA synthesis kit (Applied Biosystem). The process of cDNA synthesis was conducted based on standard procedure from the kit. Generated cDNA from all samples was then diluted to 100 ng to be used in real time PCR.

*Primer and probes used in real time PCR*

Primer and probes used in this study were designed to avoid genomic DNA amplification by spanning exon-exon junction. For PD-1, PD-L1, and GAPDH, each primer pair and its probe were formulated by Applied Biosystem into ready-to-used Custom TaqMan Gene Expression Assay. For *PD-L2*, pre-design TaqMan Gene Expression Assay Hs01057777_m1 was used. Sequences of PD-1 primers and probe are 5‘-AGGCATGCAGATCCCACA-3’ (forward), 5’-CCTGTCTGGGGAGTCTAAGA-3’ (reverse), 5’-TCTGGGCGGTGCTACAACT-3’ (probe). Sequences of PD-L1 primers and probe are 5’-GTGGCATCCAAGATACAAACTCAA-3’ (forward), 5’-TCCTTCCTCTTGTCACGCTCA-3’ (reverse), 5’-TCAAGCAGGGATTCTCAACC-3’ (probe). Sequences of GAPDH primers and probe are 5’-AGCCTCAAGATCATCAGCAA-3’ (forward), 5’-ACTGTGGTCATGAGTCCTTC-3’ (reverse), 5’-CTGCACCACCAACTGCTTAG-3’ (probe). 

*Real time PCR (qPCR)*

The real time PCR reaction contained 20 µL reaction mixture consist of 10 µL TaqMan Gene Expression Master Mix, 1 µL Custom TaqMan Gene Expression assay (primer and probe), 5 µL nuclease free water, and 4 µL cDNA. PD-1, PD-L1, PD-L2, and GAPDH reactions of each sample before and after therapy were run together in Fast 7500 Real-Time PCR System (Applied Biosystem). After 50^o^C (2 minutes) and 95^o^C (10 minutes) hold stage, the qPCR reaction was continued with 40 cycles of 95^o^C (30 seconds) denaturation and 62^o^C (1 minute) annealing and extension. The real time PCR data was analyzed using 2^-∆∆CT^ method (Livak and Schmittgen, 2001) with GAPDH as reference gene (internal control). By using 2^-∆∆CT^, we determine the fold changes of each sample. Fold changes more than 1 were categorized as increased *PD-1, PD-L1*, or* PD-L2* expression, and less than 1 were categorized as decreased *PD-1, PD-L1*, or *PD-L2* expression.

*Statistical analysis*

Statistical analysis was performed using IBM SPSS 21. Statistical comparisons between PD-1, PD-L1, and PD-L2 and clinic pathological were assessed by the Chi-Square test (*χ*^2^ test). Correlation between fold change value of PD-1, PD-L1 and PD-L2 were assessed by Spearman Correlation. Analysis between effect therapy and fold change of PD-1, PD-L1 and PD-L2 were assessed by Independent T-test. Overall Survival (OS) were estimated using the Kaplan-Meier method. Cox proportional hazards model was used to estimate the prognostic factor of PD1, PD-L1, and PD-L2 on overall survival. All analyses were hypothesis-driven by P < 0.05 was considered statistically significant.

## Results

*Patients Characteristics*

Among the 80 patients, mean of the age were 47.8 years old. A large portion of primary tumor histology was invasive ductal carcinoma, accounting for 91.3%. All patients’ histology grades were low grade (52.5%) or high grade (47.5%). Thirty-nine (48.7%) patients were in stage 3B and 41 patients (51.3%) were in stage 4. Most patients were estrogen receptor (ER) positive (68.7%), progesterone receptor (PR) positive (66.3%), and Her2 negative (70%). Breast cancer subtypes were mostly found in Luminal A (36.3%) and Luminal B types (36.3%)

*Effect of Therapy to PD-1, PD-L1, and PD-L2 Expression*

**Figure 1:**
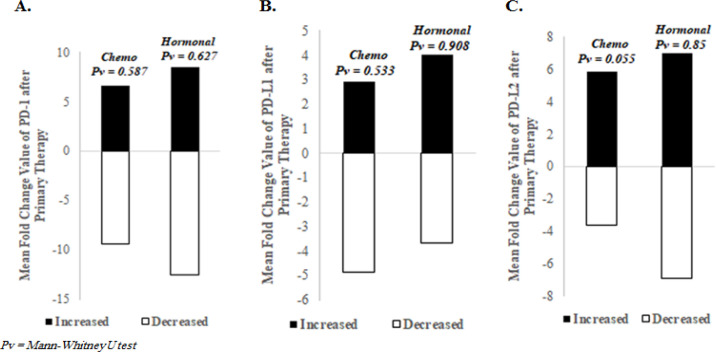
Effect of Therapy to PD-1, PD-L1 and PD-L2 Expression. (A) PD-1, (B) PD-L1, and (C) PD-L2

After undergoing chemotherapy, most breast cancer patients, 57.1% (20/35), 62.9% (22/35), and 60% (21/35) patients showed increased *PD-1, PD-L1*, and *PD-L2 *expression, respectively (mean fold change: 6.65, 2.93, and 5.88 for increased PD-1, PD-L1, and PD-L2). Meanwhile, 42.9% (15/35), 37.1% (13/35), and 40% (14/35) showed decreased *PD-1, PD-L1*, and *PD-L2* expression, respectively (mean fold change: 9.44, 4.88, and 3.65 for decreased PD-1, PD-L1, and PD-L2). Similar to chemotherapy, hormone therapy also increased *PD-1, PD-L1*, and *PD-L2* expression of most breast cancer patients. As many as 60% (27/45), 60% (27/45), and 64.4% (29/45) patients showed increased expression for *PD-1, PD-L1*, and *PD-L2* expression, respectively (mean fold change: 8.48, 4.03, and 7.02 for increased PD-1, PD-L1, and PD-L2). Meanwhile, 40% (18/45), 40% (18/45), and 35.6% (16/45) showed decreased *PD-1, PD-L1,* and *PD-L2* expression, respectively (mean fold change: 12.54, 4.03, and 7.02 for decreased PD-1, PD-L1, and PD-L2). However, the changes are not statistically significant (Figure 1).

*Relationship between PD-1, PD-L1 and PD-L2 expression*

**Figure 2:**
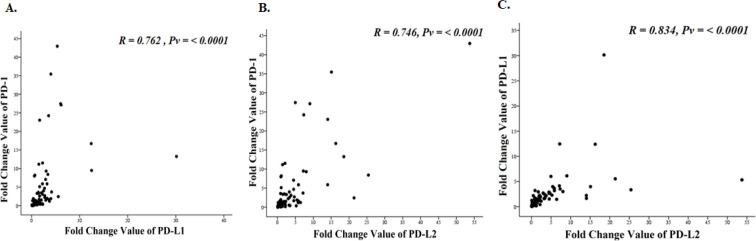
Relationship between* PD-1, PD-L1* and *PD-L2* Expression. (A),* PD-1* and* PD-L1*; (B), *PD-1* and *PD-L2*; (C), *PD-L1* and *PD-L2*

The relationship between expression changes of *PD-1 *and *PD-L1, PD-1* and *PD-L2 *and *PD-L1* and* PD-L2 *showed a significant positive correlation with a very strong close relationship (R-value: 0.762, 0.746, and 0.834 for PD-1, PD-L1, and PD-L2 respectively). We showed that samples with increased *PD-1* expression also have increased* PD-L1 *and *PD-L2* expression (Figure 2).

*Association between PD1, PDL1, and PDL2 expression after therapy and clinicopathology*

We found that the alteration of *PD-1, PD-L1* and *PD-L2* expression was not associated with age, primary tumor histology, histology grade, ER status, PR status, Her2 status, Ki67 status and molecular subtype. However, increased *PD-1, PD-L1*, and *PD-L2* expression were found more on breast cancer patients with higher ages (>40 years), ductal histology, higher grade, positive estrogen receptor (ER) status, negative HER2 status, and positive progesterone receptor (PR) status (Table 2).

*Association of PD-1, PD-L1, and PD-L2 expression with survival in breast cancer*

**Figure 3:**
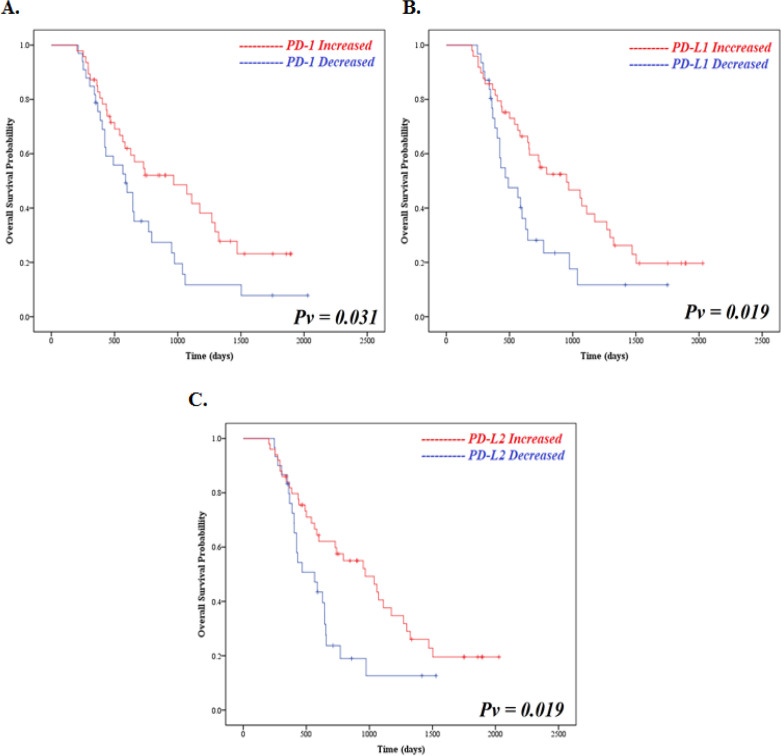
Overall Survival of PD-1, PD-L1, and PD-L2 Expression. (A) PD-1, (B) PD-L1, and (C) PD-L2

Further analysis was undertaken to explore the potential association of *PD-1, PD-L1*, and* PD-L2* with patient prognosis and survival. Kaplan-Meier survival analysis indicated that increased *PD-1, PD-L1*, and *PD-L2 *expression after primary systemic therapy were associated with statistically significant better overall survival (Figure 3). Increased *PD-1* expression was associated with longer OS than decreased* PD-1* expression in advanced breast cancer (HR=0.55, 95% CI 0.32 – 0.94; p=0.031). Increased *PD-L1* expression was associated with longer OS than decreased *PD-L1* expression (HR=0.52, 95% CI 0.30 –0.90; p=0.019). Increased *PD-L2* expression was also associated with longer OS than decreased PD-L2 expression in advanced breast cancer (HR=0.52, 95% CI 0.29 – 0.89; p=0.019). Our data identified significant association between better overall survival and increased *PD-1, PDL-1 *and *PDL-2* expression that was confirmed by multivariate analysis including prior age, stage, primary systemic therapy, histology grade, subtype and primary tumor histology (Table 3).

**Table 1:** Characteristics of Patients

Variable	N	%
Age		
Mean ±SD (year)	47.8 ± 10.65	
Range (year)	22 – 75	
Primary tumor histology		
Ductal	73	91.3
Lobular	7	8.7
Histology grade		
Low (1-2 grade)	42	52.5
High (3 grade)	38	47.5
TNM stage		
Stage 3B	39	48.7
Stage 4	41	51.3
ER status		
Positive	55	68.7
Negative	25	31.3
PR status		
Positive	53	66.3
Negative	27	33.7
Her2 status		
Positive	24	30.0
Negative	56	70.0
Subtype		
Luminal A	29	36.3
Luminal B	29	36.3
Her2 neu	14	10.0
Triple Negative	8	17.5

**Table 2:** Association between *PD-1, PD-L1, PD-L2* Expression after Therapy and Clinicopathology

Variable	PD-1		Pv	PD-L1		Pv	PD-L2		Pv
	Decreased (%)	Increased (%)		Decreased (%)	Increased (%)		Decreased (%)	Increased (%)	
Therapy									
Chemo	15 (42.9)	20 (57.1)	0.797	13 (37.1)	22 (62.9)	0.795	14 (40.0)	21 (60.0)	0.684
Hormonal	18 (40.0)	27 (60.0)		18 (40.0)	27 (60.0)		16 (35.6)	29 (64.4)	
Age									
< 40 years old	10 (50.0)	10 (50.0)	0.359	8 (40.0)	12 (60.0)	0.895	8 (40.0)	12 (60.0)	0.79
> 40 years old	23 (38.3)	37 (61.7)		23 (38.3)	37 (61.7)		22 (36.7)	38 (63.3)	
Primary tumor Histology									
Ductal	29 (39.7)	44 (60.3)	0.371	28 (38.4)	45 (61.6)	0.815	27 (37.0)	46 (63.0)	0.759
Lobular	4 (57.1)	3 (42.9)		3 (42.9)	4 (57.1)		3 (42.9)	4 (57.1)	
Histology grade									
Low	19 (45.2)	23 (54.8)	0.446	14 (33.3)	28 (66.7)	0.296	12 (28.6)	30 (71.4)	0.083
High	14 (36.8)	24 (63.2)		17 (44.7)	21 (55.3)		18 (47.4)	20 (52.6)	
TNM stage									
Stage 3B	16 (41.0)	23 (59.0)	0.968	14 (35.9)	25 (64.1)	0.61	14 (35.9)	25 (64.1)	0.773
Stage 4	17 (41/5)	24 (58.5)		17 (41.5)	24 (58.5)		16 (39.0)	25 (61.0)	
ER status									
Positive	20 (36.4)	35 (63.6)	0.188	19 (34.5)	36 (65.5)	0.252	18 (32.7)	37 (67.3)	0.191
Negative	13 (52.0)	12 (48.0)		12 (48.0)	13 (52.0)		12 (48.0)	13 (52.0)	
PR status									
Positive	20 (37.7)	33 (62.3)	0.371	17 (32.1)	36 (67.9)	0.086	18 (34.0)	35 (66.0)	0.36
Negative	13 (48.1)	14 (51.9)		14 (51.9)	13 (48.1)		12 (44.4)	15 (55.6)	
Her2 status									
Positive	11 (45.8)	13 (54.2)	0.586	11 (45.8)	13 (54.2)	0.395	11 (45.8)	13 (54.2)	0.313
Negative	22 (39.3)	34 (60.7)		20 (35.7)	36 (64.3)		19 (33.9)	37 (66.1)	
Subtype									
Luminal A	10 (34.5)	19 (65.5)	0.561	7 (24.1)	22 (75.9)	0.167	8 (27.6)	21 (72.4)	0.11
Luminal B	12 (41.4)	17 (58.6)		13 (44.8)	16 (55.2)		11 (37.9)	18 (62.1)	
Her2 neu	8 (57.1)	6 (42.9)		8 (57.1)	6 (42.9)		9 (64.3)	5 (35.7)	
Triple Negative	3 (37.5)	5 (62.5)		3 (37.5)	5 (62.5)		2 (25.0)	6 (75.0)	

Pv, Pearson Chi-Square

**Table 3:** Overall Survival (OS) by PD-1, PDL-1, PDL-2 Expression and Multivariate Analysis

Group	No.	Events	OS, Median (95% CI), days	Hazard Ratio (95% CI)		Pv^b^
				Univariate	Multivariate^a^	
PD-1 Increased	47	29	967 (511 – 1422)	0.55 (0.32 – 0.94)	0.50 (0.28 – 0.88)	0.016
PD-1 Decreased	33	27	587 (387 – 786)	NA	NA	
PD-L1 Increased	49	33	951 (590 – 1311)	0.52 (0.30 – 0.90)	0.43 (0.24 – 0.80)	0.007
PD-L1 Decreased	31	23	490 (309 – 670)	NA	NA	
PD-L2 Increased	50	33	967 (623 – 1310)	0.51 (0.29 – 0.89)	0.463 (0.24 – 0.87)	0.018
PD-L2 Decreased	30	23	566 (356 – 775)	NA	NA	

Abbreviation : NA, not applicable; ^a^ Multivariate analysis was performed to adjust for the potential effects of prior age, stage, primary systemic therapy, histology grade, subtype and primary tumor histology; ^b^ P value are from Multivariate Cox Regression analysis with adjusting for clinical covariates

## Discussion

In the present study, we have analyzed *PD-1, PD-L1, *and* PD-L2 mRNA* expression in breast cancer tissue from advanced stages patients. Our study found that expression of *PD-1, PD-L1*, and *PD-L2* in breast cancer patients is mostly increased after chemotherapy with 57.1%, 62.9% and 60% patients have an increase in *PD-1, PD-L1, *and *PD-L2* expression, respectively (Table 2). While 42.9%, 37.1%, and 40% breast cancer patients have their *PD-1, PD-L1*, and *PD-L2* expression decreased after chemotherapy. It has been explained that chemotherapy can alter the expression of *PD-1, PD-L1*, and *PD-L2 *expression in several cancer types. However, the change depends on chemotherapeutic agents and cell line that were used in the experiment (Chacon et al., 2016; Ghebeh et al., 2010; Peng et al., 2015; Zhang et al., 2008). 

Some chemotherapy agents that have been reported to increase* PD-1, PD-L1*, or *PD-L2* expression are paclitaxcel, etoposide, gemcitabine, decitabine, and cisplatin (Luo and Fu, 2016). Etoposide and paclitaxel induced *PD-L1* expression in breast cancer cell line leading to the activation of co-inhibitory signals (Zhang et al., 2008). Carboplatin–paclitaxel treatment also induced *PD-L1* expression in ovarian cancer cell lines (Peng et al., 2015). Both *PD-L1* and* PD-1* expression in leukemia cells were upregulated after decitabine treatment (Yang et al., 2013). Cisplatin could increase the expression of *PD-L1* in hepatoma H22 cells when the concentration is less than IC_50_ (Qin et al., 2010). Gemcitabine or paclitaxel was also enhanced *PD-L1 *expression in human pancreatic cell lines both in protein and mRNA level (Doi et al., 2017). Nonetheless, some chemotherapy drugs could downregulate *PD-1, PD-L1*, or *PD-L2* expression. Oxiliplatin inhibit *PD-L2* expression thus limiting immunosuppression by tumor cells and dentritic cells (Lesterhuis et al., 2011). Treatment with panobinostat suppresses *PD-1* expression in lymphoma (Oki et al., 2014). Research by Sheng et al. (Sheng et al., 2016) revealed the downregulation of *PD-L1* expression in tumor cells of NSCLC patients after treatment with neoadjuvant chemotherapy (paclitaxel, pemetrex, and TKI). After chemotherapy, positive *PD-L1* expression changed from 75% to 37.5% (Sheng et al., 2016).

In this study, 5-FAC (5-Fluorouracil, Adriamycin, and Cyclophosphamide) was used in the treatment of breast cancer patients. It has been reported that 5-Fluorouracil induce *PD-L1* surface expression on breast cancer cell lines (Zhang et al., 2008). Doxorubicine (adriamycin) is reported to upregulate *PD-L1* nuclear expression, although downregulate its surface expression in tumor. Thus, these previous finding supported our results that after 5-FAC treatment, *PD-1, PD-L1*, and *PD-L2* expression in most breast cancer patients are increased. Meanwhile, the effect of cyclophosphamide on* PD-1, PD-L1*, or *PD-L2* expression hasn’t been known. Moreover, it has been proposed that combination between 5-Fluorouracil, Adriamycin, or Cyclophosphamide with anti PD-1/ PD-L1 might give positive impact on cancer patients (Bailly et al., 2020). 

The exact mechanism on how chemotherapy work on tumor microenvironment and affect *PD-1, PD-L1, *and *PD-L2* expression is still not clear. However, some studies reported that some chemotherapeutic agents involved in several biological pathway. Chemotherapeutic agents through interferon (IFN)-γ-independent and IFN-γ-dependent may upregulate *PD-L1* expression by activating different signal such as JAK/STAT3, PI3K/AKT, RAS/RAF, or release several immune suppression cytokine (Luo and Fu, 2016). In breast cancer, signaling through key proliferative pathways, like PI3K/ AKT and MEK/ERK is known to induce *PD-L1* expression (Crane et al., 2009; Hasan et al., 2011). 

Similar with chemotherapy, the *PD-1, PD-L1*, and *PD-L2 mRNA* expression are mostly increased after patients underwent hormonal therapy. Percentages of increased *PD-1, PD-L1*, and* PD-L2* expression after hormonal therapy are 60%, 60%, and 64% respectively (Table 2). It has been reported that some hormonal therapy could induce *PD-L1 *expression in several cancer. Expression of* PD-L1* is increased in MCF7 cells (breast cancer cell line) after treatment with estrogen receptor (ER) antagonist. Treatment with tamoxifen is also increased *PD-L1* expression in mouse mammary tumor virus-polyoma middle tumor-antigen (MMTV-PyMT) breast cancer mice models (Hühn et al., 2019). It also has been shown that aromatase inhibitor (AI) therapy might increase the expression of both *PD-L1* and chemokine receptor *CCR7* in tumors (Turnbull et al., 2020; West et al., 2018). In a prostate cancer trial, enzalutamide plus pembrolizumab was associated with increased *PD-L1 *expression in tumor and dendritic cells, and increased PD-1-positive in circulating T-cells (Bishop et al., 2015; Graff et al., 2016). 

It is not clear that how hormone therapy affects *PD-1, PD-L1*, and *PD-L2* expression. Nonetheless, hormone therapy is known to change hormone level such as estrogen. It has been shown that alteration of estrogen level might alter *PD-L1* expression. A study by Huhn et al., (2019) showed that estrogen deprivation could upregulate *PD-L1* expression and triggers a wide inflammatory transcriptional program in ER+ breast cancer which includes secretion of cytokine such as as IL-6 and IFNγ that trigger the activation of the JAK/STAT pathway and TNFα that activate NF-kB signaling. Another studies showed that addition of 17β-estradiol (E2) could induce *PD-1* and* PD-L1* expression suggesting that E2 signaling might be involved in PD-1/ PD-L1 pathway (Rothenberger et al., 2018).

In this study, aromatase inhibitor (AI), tamoxifen, and GnRH analogue are given to breast cancer patients. It has been reported that AI and tamoxifen could induce *PD-L1* expression (Hühn et al., 2019; Turnbull et al., 2020; West et al., 2018). Meanwhile, the effect of GnRH analogs on *PD-L1 *expression is still unknown. However, the administration of GnRH analogs is known to decrease the concentration of circulating estrogen in premenopausal women (Huerta-Reyes et al., 2019). Moreover, it has been shown that reduction of estrogen (estrogen deprivation) could increase *PD-L1* expression (Hühn et al., 2019). This may explain how GnRH analogs induce *PD-L1 *expression.

Interestingly the alteration of* PD-1, PD-L1*, and *PD-L2* expression was associated each other. Mostly, samples with increased *PD-1* expression also increased in* PD-L1 *and *PD-L2* expression (Figure 2). Another study also revealed that there is positive correlation between the *PD-1 *and *PD-L1 mRNA* expression levels in blood samples of ITP patients (Zhong et al., 2016). Yearley et al., (2017) showed positive correlation between PD-L1 and PD-L2 protein expression in breast cancer patients. This finding suggested that chemotherapy and hormone therapy might affect the same pathway involved in the alteration of* PD-1, PD-L1,* and *PD-L2* expression.

We also found that alteration of* PD-1, PD-L1*, and *PD-L2* expression are associated with survival whereas increased PD-1, PD-L1, and PD-L2 were significantly associated with good OS, while decreased PD-1, PD-L1, and PD-L2 is associated with worse OS (Figure 3). This finding is linear with previous reports which showed that high *PD-1, PD-L1*, and *PD-L2* expression is significantly associated with good survival. Various studies have reported that *PD-1, PD-L1*, and *PD-L2* expression were associated with longer recurrence-free survival, longer disease-specific survival, longer OS, DFS, and PFS in breast cancer (Ali et al., 2015; Baptista et al., 2015; Sabatier et al., 2015.; Schalper et al., 2014; Uhercik et al., 2017; Yearley et al., 2017). The relationship between survival and *PD-L1* expression in breast cancer might indicate the presence of strong antitumor immune response mediated by TILs which leading to PD-L1 upregulation. Therefore, previous results are supported our finding. In addition, higher *PD-L1* and *PD-L2 mRNA* expression were associated with better OS to atezolizumab (anti PD-L1) in melanoma, RCC, NSCLC, and metastatic urothelial (Schmid et al., 2016; Yearley et al., 2017). 

To the best of our knowledge, this study is the first that analyzed the relationship between alteration of *PD-1, PD-L1,* and *PD-L2 mRNA* expression after primary systemic therapy with survival rate. We have shown that *PD-1, PD-L1,* and* PD-L2* expression in most samples are increased after chemo and hormonal therapy and the enhancement is associated with good survival. Since *PD-L1* expression was used to assess response to PD-1/PD-L1 checkpoint inhibitor therapy, this finding indicated reassessment of *PD-L1* expression after chemotherapy or hormonal therapy should be performed. Besides, because of high *PD-L1* expression include expression in *mRNA* level is associated with good clinical outcome of anti-PD-1/PD-L1 therapy (Patel and Kurzrock, 2015; Schmid et al., 2016), we could suggest that PD-1/PD-L1 checkpoint inhibitor therapy might improve outcome of breast cancer patients who have an increased *PD-L1* expression after completion of chemotherapy or hormonal therapy.

It has been shown that the combination of chemotherapy/hormone therapy and immunotherapy might provide effective and durable anti-tumor immune response and facilitate the clearance of the residual breast cancer cells, and reducing the percentage of patients that progress into metastatic disease (Hühn et al., 2019; Luo and Fu, 2016). Therefore, our finding may support the idea of combining chemo or hormone therapy with anti PD-1/ PD-L1. This finding also revealed that *PD-1, PD-L1,* and *PD-L2 mRNA *expression potentially could be used to predict clinical outcome of breast cancer patients. However, the limitation of this study is* mRNA* expression is not the same with protein expression due to post transcriptional modifications. Thus, further study to compare *PD-L1 mRNA* expression with PD-L1 protein expression using IHC as gold standard is needed to confirm this finding.

In conclusion, Expression of* PD-1, PD-L1*, and *PD-L2* majorly increased after primary systemic therapy. Increase in* PD-1, PD-L1*, and* PD-L2* expression after therapy was significantly associated with good OS. Strong positive correlation between PD-1, PD-L1, and PD-L2 alteration after systemic therapy suggested chemotherapy or hormonal therapy may affect the same pathway to alter *PD-1, PD-L1*, and *PD-L2* expression. Our finding implied reassessment of *PD-L1* expression and the potential benefit of anti-PD-1/PD-L1 therapy after completion of systemic therapy. This finding also revealed the potential to measure* PD-1, PD-L1*, and *PD-L2 mRNA* expression to predict clinical outcome of advanced stages breast cancer patients. However, subsequent study by comparing *mRNA* expression to PD-L1 IHC is needed to confirm the result.

## Author Contribution Statement

R.K and M.A.A designed the study and took the lead in writing the manuscript. M.A.A carried the experiment and performed the measurement. F.S contributed to sample preparation. Y.P performed statistical analysis; All authors discussed the results and contributed to the final manuscript.

## Data Availability

The datasets are not publicly available due to ethical restrictions, but are available from the corresponding author on reasonable request.
